# Targeting Histone Epigenetic Modifications and DNA Damage Responses in Synthetic Lethality Strategies in Cancer?

**DOI:** 10.3390/cancers14164050

**Published:** 2022-08-22

**Authors:** Pedro A. Lazo

**Affiliations:** 1 Molecular Mechanisms of Cancer Program, Instituto de Biología Molecular y Celular del Cáncer, Consejo Superior de Investigaciones Científicas (CSIC), Universidad de Salamanca, 37007 Salamanca, Spain; pedro.lazo@csic.es; 2 Instituto de Investigación Biomédica de Salamanca-IBSAL, Hospital Universitario de Salamanca, 37007 Salamanca, Spain

**Keywords:** chromatin kinase, lysine methylase, lysine demethylase, lysine acetylase, lysine deacetylase

## Abstract

**Simple Summary:**

Dynamic chromatin remodeling is regulated by different epigenetic modifications of histones to adapt chromatin to specific cellular functions. Targeting histone epigenetic enzymes will interfere with the correct mechanisms of DNA repair. Therefore, targeting epigenetic enzymes is a potential novel strategy for synthetic lethality to facilitate tumor cell death in response to current genotoxic treatments.

**Abstract:**

Synthetic lethality strategies are likely to be integrated in effective and specific cancer treatments. These strategies combine different specific targets, either in similar or cooperating pathways. Chromatin remodeling underlies, directly or indirectly, all processes of tumor biology. In this context, the combined targeting of proteins associated with different aspects of chromatin remodeling can be exploited to find new alternative targets or to improve treatment for specific individual tumors or patients. There are two major types of proteins, epigenetic modifiers of histones and nuclear or chromatin kinases, all of which are druggable targets. Among epigenetic enzymes, there are four major families: histones acetylases, deacetylases, methylases and demethylases. All these enzymes are druggable. Among chromatin kinases are those associated with DNA damage responses, such as Aurora A/B, Haspin, ATM, ATR, DNA-PK and VRK1—a nucleosomal histone kinase. All these proteins converge on the dynamic regulation chromatin organization, and its functions condition the tumor cell viability. Therefore, the combined targeting of these epigenetic enzymes, in synthetic lethality strategies, can sensitize tumor cells to toxic DNA-damage-based treatments, reducing their toxicity and the selective pressure for tumor resistance and increasing their immunogenicity, which will lead to an improvement in disease-free survival and quality of life.

## 1. Introduction

Cancer treatment is evolving towards the application of personalized therapies. In this context, alternative drug combinations based on tumor characteristics are a likely trend. The knowledge of the pathways implicated in cancer developments, progression, metastasis and treatment resistance can be the basis for development of combined therapies based on synthetic lethality strategies. A significant push in this direction is a consequence of the identification by genomic studies of specific gene mutations and expression patterns in different tumor types and stages, which can permit to adapt treatments to the individual tumor characteristics [1,2,3]. However, detectable metastasis and their location are the consequence of selection and reflect the adaptation to the new environment and not the original metastatic cell, which might have been silent for several years before its reactivation and growth. 

In this context, hypersensitization to well-known drugs, such as those causing DNA damage, might have a common effect independent of location, stage or tumor type and in which the manipulation of chromatin can play a major role by increasing the tumor cell sensitivity to them by interfering with the repair process and the restoration of chromatin back its normal state.

Cancer treatments rely on the use of drugs causing DNA damage that have severe toxicity and side effects, such as doxorubicin, etoposide, cisplatin among genotoxic drugs, as well as radiotherapy. All of them are still mainstay treatments despite the development of many new therapies in the context of personalized strategies. Initially, synthetic lethality strategies were based on the combination of a mutation with a drug targeting a different pathway in the tumor. This is exemplified by the approach that takes advantage of DNA damage response (DDR) defects already present in tumors cells, such as *BRCA1* or *BCRA2* mutations in ovarian, breast, pancreatic and prostate cancers [4,5,6] or *IDH* mutations in gliomas [7] and combine them with new drugs, as exemplified by olaparib [8]. 

However, an alternative to mutations is to use a second drug that will functionally mimic the mutation effect in a combination of synthetic lethality [9]. Combination treatments, based on synthetic lethality, can become a potentially successful approach, since they might permit using drugs at lower concentration, reducing their toxicity and selective pressure that leads to resistance. In this context, drugs that alter chromatin dynamics or impair DNA repair pathways can hypersensitize cells to the several commonly used genotoxic treatments.

Synthetic lethality therapies can facilitate tumor cell death and increase the tumor mutational burden, which results in more potential epitopes presented by surviving tumor cells and trigger immune responses during tumor progression [3,10,11]. Tumors with a high mutagenic load are more susceptible to treatments based on immunotherapy [12,13,14], and the exploitation of these two factors can improve the success of such treatments. All these effects can lead to more effective treatments resulting in a better quality of life and longer disease-free or survival periods with a better quality of life. However, there are feasible but untested, target combinations that have a significant therapeutic potential once developed.

## 2. Synthetic Lethality and Cancer

Synthetic lethality strategies facilitate a larger diversity in the combination of druggable targets in different signaling pathways, such as those involved in chromatin remodeling and DNA damage responses (DDR) whose inhibition opens up the possibility of alternative therapeutic options to improve cancer treatment.

**Figure 1:**
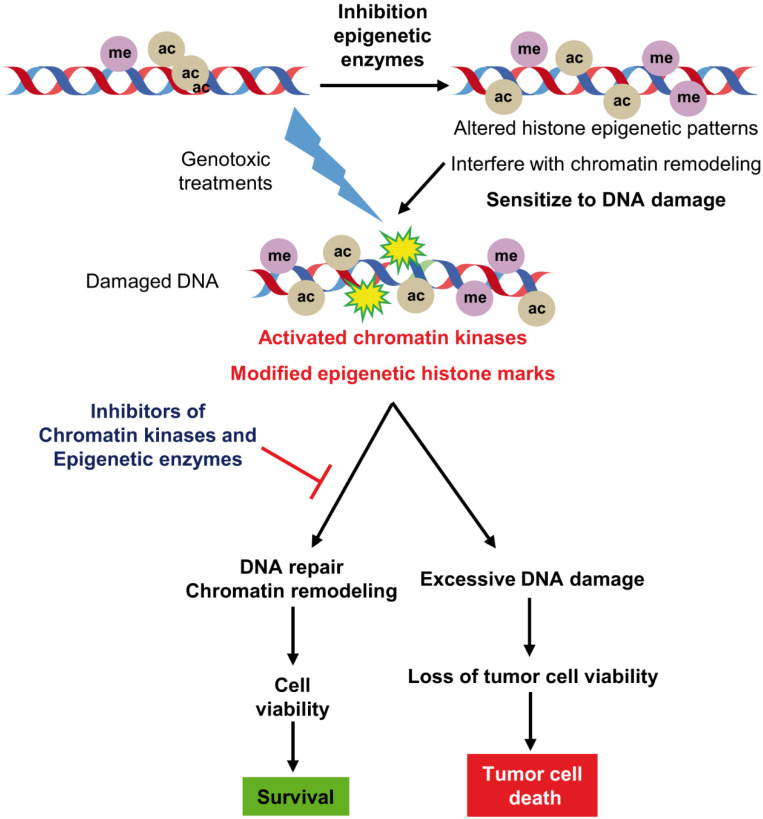
Targeting of the chromatin epigenetic enzymes and nuclear kinases that can modulate the response to therapies based on DNA damage. Inhibition of histone epigenetic modifications cam facilitate DNA damage by facilitating accessorily to DNA genotoxic treatments and by impairing the dynamic chromatin remodeling associated with specific DNA repair mechanisms. ac: acetylation. me: methylation.

DNA damage activates several kinases in specific DDR pathways depending on the type of DNA lesions [15,16] and also alter the local epigenetic modifications of histones (Figure 1). Epigenetic changes modify the biological properties of the tumor cell by altering gene expression and can promote either cell differentiation, growth arrest or an impairment of DDR pathways (Figure 1). Therefore, altering the tumor cell epigenome impairs chromatin dynamics required for DNA damage recognition or DDR progression [17] and can facilitate the sensitivity to genotoxic treatments [10,11].

Pathways regulating the dynamic chromatin organization underlies the sensitivity of tumor cells to DNA-damage-based cancer treatments. Among these pathways are those associated with chromatin remodeling, as well as those associated with initiation or progression of specific DNA damage responses. The targeting of chromatin has several layers, from chromatin organization, regulators of chromatin, chromatin remodelers and specific DNA repair pathway proteins. 

Covalent modifications of histones regulate chromatin organization and includes methylation, acetylation and phosphorylation that may alter the balance between methylation and acetylation in common or different histone lysine residues. Therefore, pharmacological manipulation of any of these five types of enzymes can alter tumor cells sensitivity to different types of genotoxic treatments and can have a significant potential for development of novel synthetic lethality strategies for specific cancers.

This is not only based on DNA damage but also likely by altering gene expression and differentiation of tumor cells. In this context, there is evidence indicating that targeting epigenetic enzymes can be useful in cancer treatment [18]. All of them offer opportunities for identification of new vulnerabilities in cancer cells.

## 3. Epigenetic Chromatin Remodeling as Pharmacological Target

The pathogenesis of cancer implicates alterations in chromatin epigenetic marks [19]. The inhibition of signaling pathways associated with the regulation of chromatin remodeling is a potential strategy of synthetic lethality. Particularly by targeting histone epigenetic enzymes, such as acetylases and deacetylases, histone methylases and demethylases or by targeting kinases located on chromatin that coordinate its organization and associated functions. 

The alternative epigenetic modifications, in several histone lysine residues, generate many different alternative epigenetic combinations in individual histone tails and in nucleosomes that determine their effect at specific locations in chromatin [20]. These epigenetic enzymes belong to large protein families with hundreds of members in different subfamilies. For most of these enzymes their pattern of expression in different cell types and their association with specific biological functions and cell types is unknown. However, these protein families are druggable and thus can be exploited for therapeutic purposes, once the tumor type in which they function in this context are identified.

All of these enzymes are druggable targets and some have inhibitors with potential for clinical applications. Histone functional roles are modified by different combinations of lysine methylations and acetylations and form a network of epigenetic modifications in which context and location on chromatin regions determine their role in different biological processes that require a dynamic chromatin remodeling, among which are transcription, replication, recombination or DNA damage responses (DDR) [21].

**Figure 2:**
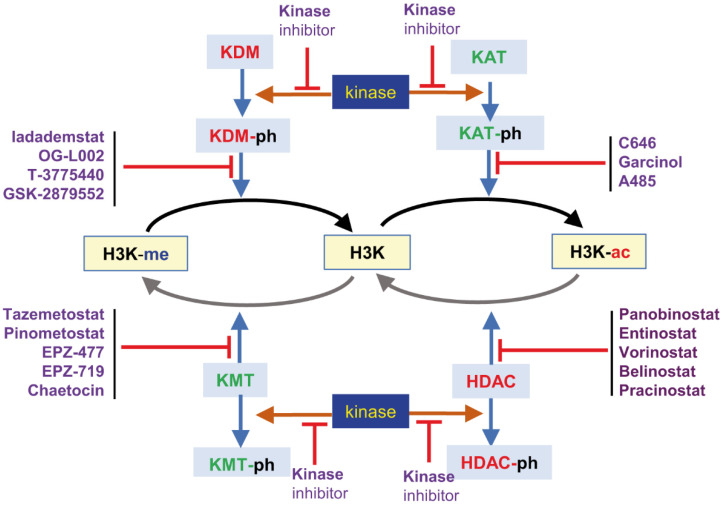
Alternative covalent modifications, methylation or acetylation of histone H3K in different residues, by enzymes mediating the alternative epigenetic modifications, the effect of regulatory phosphorylation coordinating the modifications and drugs targeting different epigenetic enzyme activities. These epigenetic drug combinations can vary depending on the specific lysine residue modified in individual H3 histone tails or in combination with additional modifications in any of the other histones in the nucleosome. Therefore, there is a very large number of potential combinations that need to be identified and tested in specific tumor types or stages.

The restoration of damaged DNA back to normal requires a dynamic remodeling of chromatin, which is associated with several epigenetic modification of histones (Figure 2). Both acetylation and methylation can have dual roles by either the activation or inhibition of chromatin-associated functions, such as transcription, replication or DNA repair (Figure 2). These epigenetic modifications occur in several lysine different histone; however, some residues can be affected by alternative modifications, methylation and acetylation, which requires a coordination of the four types, of two or four epigenetic, enzymes. (Figure 2), likely mediated by their phosphorylation in Ser or Thr by nuclear/chromatin kinases. 

In cancer, there are tumors associated with mutations or amplification of genes that code for specific lysine modifications of histones [22,23,24,25], further supporting their potential role as therapeutic targets (Figure 2). For several members of these enzymes there is evidence pointing to their therapeutic potential, which is still far from being an established treatment and novel preclinical, and later clinical studies are needed to determine their clinical usefulness and identify their specific tumor indication.

## 4. Histone Methylation 

### 4.1. Targeting Lysine Methyl Transferases

Lysine methyl transferases (KMT) play different roles in chromatin biology and disease [26] and has been the more successful in the identification of new treatment approaches. There are two groups of lysine methyltransferases. The members of the first KMT group are characterized by proteins that have a SET domain and is composed by fifty-five proteins in humans; however, only half of them are KMT active [26]. The 7βS family forms the other KMT group that has 150–160 members in humans [26]. 

However, for most lysine demethylases (KDM), their specificity is unknown regarding the individual KMT expression in different cell types, their specific protein targets and their role in different biological contexts, all of which can be exploited for therapeutic purposes when known [27]. For several KDM and KMT inhibitors there is evidence supporting that they are potential candidates for synthetic lethality (Table 1).

There is some evidence pointing to the potential role of KMTs as targets. DOT1L (KMT4) is the only non-SET domain KMT that regulates H3K79 mono-, di- and tri-methylation and is required for chromatin relaxation and a correct DNA damage response [28]. DOT1L plays a relevant role in the pathogenesis of mixed lineage leukemia (MLL) and thus is a potential therapeutic target. The inhibitor pinometostat (EPZ-5676) is effective as single agent in tumors with MLL rearrangements [29]; however, its combination with azacytidine, a hypomethylating drug or daunorubicin enhances its therapeutic effect [30]. Moreover, the use of EPZ-4777 is more effective when used in combination with SRT1720, a potent Sirt1 agonist [31]. 

EZH2 (KMT6A) mediates the trimethylation of H3K27 (H3K27me3) and is overexpressed in many types of cancer, including prostate, kidney, breast and lung in which it promotes cell migration, colony formation and genomic instability [32]. Tazemetostat is an inhibitor of EZH2 that blocks H3K27 methylation. Tazemetostat sensitizes cells to these genotoxic treatments by facilitating the accumulation of excessive DNA damage and leads to their death [33]. 

Tazemetostat induces the expression of CCL17 in B-cell lymphoma lines and enhances T-cell recruitment [34], indicating a potential role in anti-tumor immune responses that may need to be explored in the context of immunotherapies. Tazemetostat has been approved for epithelioid sarcoma treatment [35], refractory follicular lymphoma [36]. EZH2 is overexpressed in pediatric acute monocytic leukemia and the GSK126, UNC1999 and EPZ-5687 inhibitors suppresses the EZH2 activity on H3K27 leading to a reduction of proliferation and increased apoptosis. Treatment of pediatric AML subtypes with these EZH2 inhibitors was enhanced when combined with selinexor, an inhibitor of XPO1, a nuclear export protein [37]. 

Another interesting combination is the targeting EZH2 with tazemetostat that hyper sensitizes ovarian cancer cells by promoting the NHEJ pathway and causing chromosomal abnormalities and mitotic catastrophe in HR-proficient cells treated with olaparib [38]. Olaparib, and its analogs, is a PARP (poly ADP-ribose polymerase) inhibitor that is already in clinical use and will not be further reviewed [8]. EZH2 resistance to mutants that facilitated the accumulation of H3K27me3 was detected in a model of diffuse large B-cell lymphoma (DLBCL) [39]. EZH2^C663Y^ and EZH2^Y726F^ mutants were resistant to inhibition by GSK126 or EPZ-6438 but sensitive to UNC1999 [40]. 

This indicated that, by changing the inhibitor used, the tumor cell response can be manipulated and be exploited for adjustment of treatment to the individual case situation. In an experimental model using sarcoma cell lines, tazemetostat impairs the formation of H4K20me2, mediated by SUV4-20H (KMT5A) and needed for recruitment of 53BP1 to locations with DNA damage induced by treatment with either doxorubicin or ionizing radiation [33,41].

H3K36 methylation is highly specific, and its dimethylation is performed by NSD1/2/3 (KMT3B/G/F), and its trimethylation by SETD2 (KMT3A), which is not dependent on a previous H3K36me2 by NSD. Therefore, these different levels of H3K36 methylation represent two different chromatin locations and roles [42]. However, despite NSD2 as a potential as therapeutic target, there are no potent and selective inhibitor to date [43]. 

The overexpression of NSD2 in multiple myeloma is a consequence of its regulation by a strong IgH enhancer in the *NSD2* gene, and this NSD2 overexpression impairs the binding of EZH2 and the reprogramming of the myeloma epigenome because of locally altering the H3K36 and H3K27 methylation patterns [44]. H3K36 dimethylation (H3K36me2) by NSD2 is sufficient for gene activation [45]. Therefore, targeting NSD2 with specific inhibitors could become part of a potential anti multiple myeloma therapy, which by impairing its interaction with SRC-3 (steroid receptor coactivator-3) facilitates overcoming the resistance of multiple myeloma to bortezomid, a proteasome inhibitor [46]. 

H4K20 dimethylation (H4K20me2) is mediated by NSD2 (KMT3G, MMSET, WHSC1) and is associated with the DNA damage response mediated by the Non-homologous end joining (NHEJ) [41] and nucleotide excision repair (NER) [47] pathways, playing a role in the selection of the NHEJ pathway to repair DNA double-strand breaks [41,48]. NSD2 inhibition with tazemetostat or chaetocin, two KMT inhibitors, sensitizes cells to doxorubicin or ionizing radiation in leiomyosarcoma and osteosarcoma cell lines [33]. 

SETD2 (KMT3A) exclusively mediates the trimethylation of H3K36 (H3K36me3), a chromatin mark of transcriptional elongation. H3K36me3 is required for the activation of ATM in DNA double-strand breaks, in which it participates in preparing the local chromatin organization for the repair process [49], and there is a cross talk with H4K16ac [50], mediated by Tip60 that also regulates the acetylation and activation of ATM [51,52]. Therefore, the targeting of SETD2 is a potential candidate for combination therapies with Tip60 or ATM inhibitors, which can become novel therapeutic strategies for cancer treatment. Recently, a fist-in-class inhibitor, EPZ-719, was reported and is in preclinical validation studies [53]. 

The SETDB1 (KMT1E) methyltransferase trimethylates H3K9 (H3K9me3), a mark associated with gene silencing in combination with DNA methylation [54]. H3K9me3 mediated by SETDB1 contributes to the repression of developmental genes that maintain cells in an undifferentiated state [55,56], an important component for the metastatic potential of tumor cells. SETDB1 overexpression in tumors is associated with immune exclusion and resistance to immune checkpoint blockade and in lung cancer can function as an oncogene [57]. The silencing of SETDB1 reactivates the expression of immune stimulatory genes and triggers an anti-tumor cytotoxic T-cell response in a murine model [58]. Thus, the pharmacological targeting of SETDB1 can increase the tumor cell response to immunotherapies. It would be interesting to test the combination of SETDB1 and DNMT inhibitors in this context.

G9A (KMT1C) mediates H3K9 methylation and is emerging as an epigenetic target in melanoma [59]. The dual inhibition of G9A and EZH2 stimulates an anti-tumor immune response in high-grade serous ovarian carcinomas [60]. The roles of G9A associated with cancer stemness indicate that it is an interesting target for development of specific inhibitors with potential applications in cancer treatment [61], which might prevent or delay, tumor relapses.

**Table 1:** Candidate combinations for synthetic lethality strategies targeting chromatin methylases (KMT) and demethylases (KDM).

Target Combinations		Inhibitor Combinations	Tumor	Ref.
1	**NSD2 (KMT3G)**	SRC-3	Multiple myeloma	[46]
	Proteasome	bortezomid		
2	**NSD2 (KMT3G)**	INCB054329	Multiple myeloma	[62]
	JAK1	itacitinib		
3	NSD2 (KMT3G)	Tazemetostat	Osteosarcoma, leiomyosarcoma cell lines	[33]
	DNA intercalation/Topoisomerase I inhibition	Doxorubicin		
4	**EZH2 (KMT6A)**	Tazemetostat	Osteosarcoma, leiomyosarcoma cell lines	[33]
	DNMT	Azacitidine/Decitabine		
5	**DOT1L (KMT4)**	Pinometostat (EPZ-5676)	MLL rearrangements	[29]
	Topoisomerase I inhibitors, DNA methylation inhibitor	daunorubicin Azacytidine		[30]
6	**DOT1L (KMT4)**	EPZ-4777	MLL rearrangements	[31]
	Sirt1	SRT1720		
7	**SETD2 (KMT3A)**	EPZ-719	Preclinical	[53]
8	**SETDB1 (KMT1E)**	mithramycin	Preclinical	[53,57]
	DNA methylation	Azacytidine		[54]
9	**G9A (KMT1C)**	HKMTI-1-005	Melanoma, ovarian carcinoma	[59,60]
	EZH2			
10	**KMT**	chaetocin	NSCLC cell lines	[63]
	DNA damage	IR, doxorubicin		
11	**KMT**	chaetocin	Hepatoma and sarcoma cell lines	[33,64]
	Autophagy/Atg5	Bafilomycin A1		
12	**KMT**	Tazemetostat, chaetocin	Osteosarcoma, leiomyosarcoma lines	[33]
	**DNA damage**	doxorubicin		
13	**LSD1 (KDM1A)**	ORY-1001 (iadademstat)	AML	[65]
14	**LSD1 (KDM1A)**	ORY-1001 (iadademstat)	Luminal B and HER2 amplified breast cancer	[66]
15	**LSD1 (KDM1A)**	T-3775440	Small cell lung carcinoma (SCLC)	[67]
16	**LSD1 (KDM1A)**	GSK2879552	SCLC cell lines	[68,69]
17	**LSD1 (KDM1A)**	tranylcypromine GSK2879552	Acute myeloid leukemia (AML)	[70,71]
	MEK1	trametinib		
18	**LSD1 (KDM6)**	Corin dual inhibitor	diffuse intrinsic pontine glioma (DIPGs)	[72]
	HDAC1A			

### 4.2. Targeting Lysine Demethylases (KDM)

Targeting KDM has received less attention than KMT. However, there are some promising new drugs and depletion experiments indicating they can be of use in some tumors [68,70]. Methylation of histones H3 and H4 in several lysine are associated with different functional roles. Thus, H3K9m3 is associated with enhancer activation in cooperation with H3K27ac. Overexpression of LSD1 (KDM1A) occurs in many tumor types [68]. LSD1 demethylates H3K4m3 and K3K9me and regulates anti-tumor immune responses [73]. Depletion of LSD1 enhances tumor immunogenicity, facilitates T cell infiltration and causes a significant response in melanomas that are refractory to anti-PD-L1 blockade [73]. 

In a murine LSD1 knockout, modelling melanomas, there is an enhancement of tumor immunogenicity and T-cell infiltration, which suggest that inhibition of LSD1 can facilitate the effect of immunotherapy [73]. Targeting LSD1 with the T-3775440 inhibitor has been partially effective in the inhibition of SCLC cell proliferation and retarded tumor growth by disrupting its interaction with SNAD domain proteins [67]. LSD1 binds to and suppresses the *NOTCH1* locus expression. The inhibition of LSD1 with iadademstat (ORY-1001) reactivates *NOTCH1* signaling in a chemo resistant PDX model of small cell lung cancer (SCLC) [74]. 

GSK2879552, an irreversible inhibitor of LSD1, inhibited growth of SCLC and AML cells; however, the effect was mainly cytostatic. GSK2879552 prevented growth of xenografted SCLC cells, and its effects correlated with a DNA hypomethylation pattern, which could become a potential biomarker of sensitivity to this drug [69]. An additional effect of LSD1 inhibition with GSK2879552 is the hypersensitization it causes to MEK inhibitors in AML cells, in which the activation of the MEK pathway is a mechanism of resistance to LSD1 inhibitors [71]. 

The LSD1 (KDM1A) inhibitor iadademstat (ORY-1001) induces the accumulation of H3K4me2 in its target genes and is currently in clinical trials [65]. Iadademstat (ORY1001) reduces the leukemic stem cell capacity in acute myeloid leukemia (AML), by inducing blast differentiation and extending survival in a PDX model [65]. Iadademstat can facilitate differentiation of acute myeloid leukemia (AML) cells [75] and also stimulates anti-tumor immunity enabling checkpoint blockade [73]. Iadademstat targets Sox2-driven breast cancer stem cells and can be a candidate for epigenetic therapies in luminal-B and HER2 positive breast cancer [66]. 

The H3K27M mutation cause an epigenetic dysfunction frequently detected in diffuse intrinsic pontine gliomas [72]. Corin is a dual inhibitor against LSD1/HDAC1A and induces a chromatin reprogramming that increases H3K27m3, which was suppressed in H3K27M mutants and simultaneously increases H3K27ac and H3K27me1 in genes associated with differentiation. Corin induces cell cycle arrest, differentiation and tumor cell death, which improves the patient survival time [72]. It is unknown whether combination of specific inhibitors for each of these two enzymes, LSD1 and HDAC1A, will lead to a similar result. The loss of KDM6 enhances the sensitivity of cells to EZH2 inhibitors, thus both cooperate in impairing the trimethylation of H3K27 and facilitate chromatin relaxation [76], which can make cells more sensitive to DNA damage.

Targeting members of the Jumonji family, the other KDM family, has so far received less attention from a pharmacological perspective, and this family has the potential problem of their specificity.

## 5. Targeting Histone Acetylases

Histone acetylation in general has been associated with chromatin relaxation and opening to facilitate different biological processes including gene transcription, replication and DNA damage responses [77]. Thus, alteration of acetylation can alter gene expression patterns as well as DNA repair mechanisms that requires an initial local chromatin relaxation. Histone acetylation is regulated by two families of enzymes histone (or lysine) acetylases (HAT or KAT) and histone (lysine) deacetylases (HDAC). Some of the KAT and HDAC inhibitors that are potential candidates for synthetic lethality are shown in Table 2.

### 5.1. Targeting Lysine Deacetylases

Histone deacetylases (HDAC) influence DNA damage signaling and DNA repair by modifying the relaxation of chromatin [83]. There are eighteen human HDAC proteins that are classified in four different classes [84]. Type 1 includes HDAC 1, 2, 3 and 8. Type IIa: HDAC 4, 5, 7 and 9; type IIb: HDAC 6 and 10. Type III: Sirt1 and Sirt2 are a different group and the deacetylation mechanism is NAD dependent. Type 4: HDAC 11. However, the expression pattern of these HDAC in different cell types is unknown. There are several HDAC inhibitors among which are selisistat (Sirt inhibitor), entinostat, vorinostat and panobinostat targeting several different HDAC, which have a limited substrate specificity [84].

Dysregulation of HDAC alters the cellular proteome and consequently their cellular functions [85]. HDAC overexpression is associated with poor prognosis in several types of tumors [84,86]. HDAC overexpression facilitates drug detoxification by increasing levels of glutathione that eliminate toxic drugs, such as cisplatin in squamous cell carcinomas [87] and doxorubicin in colorectal and lung cancer [88]. In this context, HDAC inhibitors sensitize tumor cells to these therapeutic drugs by impairing their detoxification and facilitating their anti-tumor effects [88].

The knockdown of HDAC 1 and 2 in glioblastoma cells reduces tumor cell proliferation [89,90,91,92] suggesting that these enzymes can modulate the chromatin epigenetic marks. The inhibition of deacetylation facilitates chromatin relaxation, which is more susceptible to undergo DNA damage, a characteristic that can be exploited to sensitize tumor cells to genotoxic treatments. Histone acetylation can alter gene expression patterns and affect differentiation and lead to increased immunogenicity and cell death. 

In general, HDAC inhibitors have a minor effect on promoter acetylation [93] but have a significant impact in facilitating the H3K27 trimethylation (H3K27me3) associated with silencing of enhancer sequences [94]. In some particular contexts, such as those associated with DNA damage targeting strategies, the inhibition of HDAC sensitizes neuroblastoma cells to etoposide, melphalan or carboplatin [95] and melanoma cells to temozolomide [96].

More novel and specific inhibitors of HDAC can also cooperate with the inhibition of other chromatin proteins, such as PARP, associated with DNA repair [97,98]. Panobinostat, an HDAC inhibitor, enhances olaparib efficacy in a model of ovarian cancer by increasing DNA damage, reducing cell proliferation and enhancing T-cell infiltration [78]. Panobinostat in combination with other drugs, such as proteasome inhibitors, immunomodulatory drugs and monoclonal antibodies, have shown potential in the treatment of multiple myeloma [79]. 

Panobinostat or vorinostat in combination with OTX015, a bromodomain inhibitor preventing reading histone acetylation and resulting in a double interference with chromatin regulators, causes a loss of cell viability and increases apoptosis in different glioblastoma cells, as well as in an orthotopic model of glioblastoma [80]. Panobinostat in combination with CBL0137, targeting the FACT (Facilitates Chromatin Transcription) complex, induces an interferon response and has shown preclinical efficacy in neuroblastomas [99].

The combination of HDAC inhibitors with immunotherapy is a promising therapeutic strategy [100,101]. An important effect of using HDAC inhibitors is that they facilitate the response to different immune therapies. Entinostat and vorinostat in combination with anti-PD1 or anti-PD-L1 antibodies, improve the immune response in clinical trials [81,100]. It is likely that these responses will further improve with newer inhibitors, such as panobinostat.

Sirt1 is a deacetylase of histones H1, H3 and H4 that regulates chromatin remodeling [102] and base excision repair [103]. Sirt1 is overexpressed in several types of cancers, such as colon, prostate, breast, liver sarcomas, leukemias and lymphomas [104]. Sirt1 confers chemoresistance in lung cancer by deacetylating and stabilizing XRCC1 [105]. Therefore, Sirt1 inhibition in therapeutic combinations with cisplatin or adriamycin reduces chemoresistance in lung cancer cells [105]. 

The inhibition of Sirt1 with JGB1741 induces apoptosis in different tumor cells lines but at concentrations that are not of clinical use [106]. Moreover, Sirt2 inhibitors have shown some antitumor effects, such as the induction of p53-dependent apoptosis non-small lung cancer cells [107]. Other HDAC inhibitors facilitate the degradation of c-Myc in several tumor types driven by this oncogene [108].

### 5.2. Targeting Lysine Acetyl Transferases (KAT)

Histone acetylation is a major modification implicated in processes requiring a dynamic chromatin remodeling for specific functions, such as transcription or DDR. However, targeting KAT (lysine acetyl transferases) has not received as much attention as HDAC inhibitors. The KAT family is composed of seventeen members grouped into several families and defined by the conservation of their HAT domain. Several KATs play a relevant role in the regulation of DNA damage responses, particularly by members of the MYST family [109,110]. 

This family has conserved three defined components, an acetyl-CoA binding site, a zinc finger and a helix-turn-helix motif that form the catalytic domain. Among them, the Tip60/KAT5 protein is the most promising candidate for therapeutic targeting. The Tip60 knockout is lethal [111], and downregulation of Tip60 in tumor cells causes cell death [112,113]. Tip60 is also implicated in resistance to cisplatin [114,115]. Tip60 knockdown impairs ATM activation and sensitizes cells to irradiation [116]. In many types of cancer, Tip60 levels are reduced, indicating a certain level of activity is necessary for survival, and a further reduction in levels lead to tumor cell death [110].

There is data indicating that KAT inhibitors, mainly centered on the targeting P300 and CBP (CREB-binding protein) acetyl transferases, might be useful in some tumors. In melanomas with the BRAF (V600E) mutation that are resistant to BRAF inhibitors, treating tumor cells with C646, a p300 inhibitor, overcame the resistance to BRAF inhibitors. This effect is mediated by CRAF and ATAF and prevented the acetylation of NONO [82]. C646 inhibits tumor growth of pancreatic cancer [117], gliomas [118] and gastric cancer cell lines [119]. However, there might be specific tumor types and specific tumor cell contexts, in which KAT might be suitable targets, for example by impairing H3K27 acetylation associated with enhancers that are needed for cell viability.

Another potential cooperating approach can be based on the targeting of kinases, required for activation of KATs, as it might be the case of VRK1, a nuclear kinase that phosphorylates Tip60/KAT5 leading to its stabilization and translocation to chromatin, where it mediates the H4K16 acetylation [52], needed for progression of the DNA damage response [16].

## 6. Targeting Nuclear Kinases Associated with Chromatin Remodeling and Damage Response Pathways

The DNA damage response mechanisms involve a dynamic remodeling of chromatin that affect several sequential processes, including detection of DNA damage, identification of its type, preventing degradation of free DNA ends, recruitment of specific repair enzymes and restoration back to normal chromatin organization, which are coordinated by kinases. Nuclear kinases are implicated in regulation of chromatin remodeling processes ranging from cell cycle progression to transcription, replication or DNA damage responses. Therefore, these kinases are potential therapeutic targets. 

**Figure 3:**
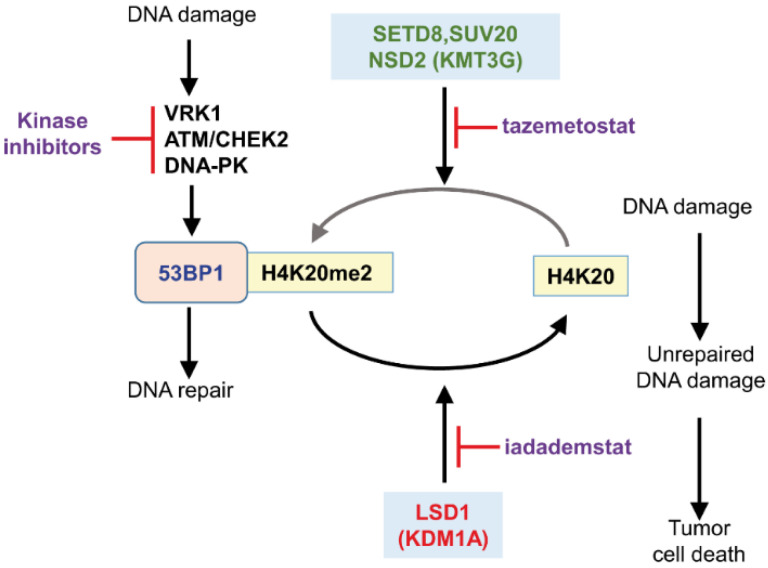
Effect methylation and demethylation inhibition on histone H4 in K20 and drugs that can alter the DNA damage response mediated by non-homologous end-joining pathway for which the dimethylation of H4K20 is required for recruitment of 53BP1.

**Figure 4:**
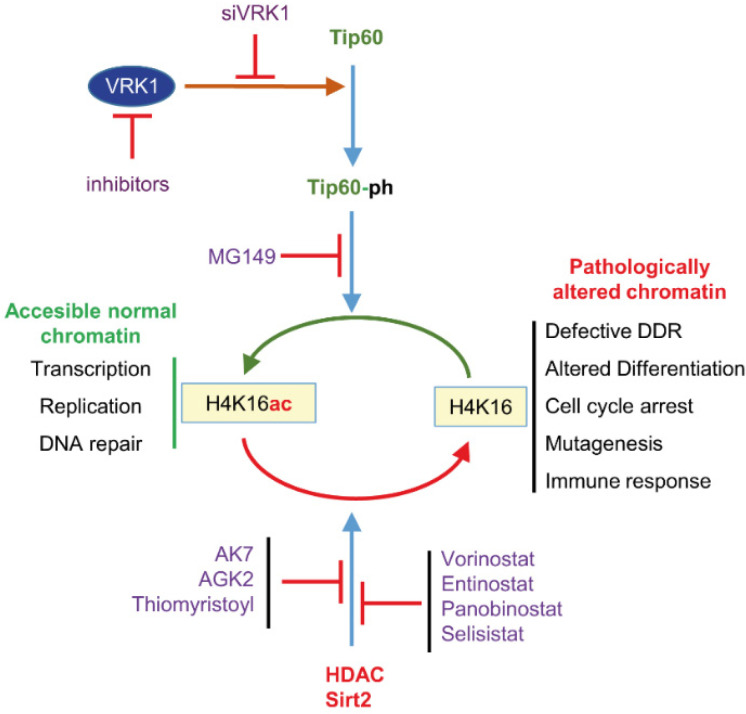
Effect of epigenetic inhibitors on H4K16 acetylation and deacetylation that modulate chromatin relaxation associated with different roles in transcription, replication and DNA damage, some of which are local o gene specific, and others affect chromosomes more globally. Therefore, there is a very large number of potential combinations that need to be identified and tested in specific tumor types or stages.

Among the nuclear kinases that can be targeted are Aurora, haspin, ATM, ATR, DNA-PK and VRK1 (Table 3). All of them, by either mutation or overexpression, are associated with the tumor phenotype, and some of them have specific inhibitors. However, these kinases in general have received little attention in the context of synthetic lethality strategies. Drugs targeting all these steps implicating chromatin reorganization can sensitize tumors cells to DNA damage therapies and facilitate tumor cell death (Figure 3 and Figure 4). This effect can be enhanced in tumors that already have mutations in mismatch repair genes display and enhanced immunogenicity [120,121].

Drugs that cause DNA damage or ionizing radiation constitute two basic cancer treatments used in many protocols for many types of cancers. Either the generation of excessive DNA damage in tumor cells, by interfering with the role of topoisomerase I, a consequence of DNA intercalation by anthracycline drugs or by oxidative stress is the molecular effect of these treatments. The accumulation of excessive DNA damage will cause tumor cell death. 

Recently, the footprint of epigenetic marks detected a pattern of DNA lesions in the form of insertions and deletions caused by topoisomerase I and specific defective DNA repair mechanisms, suggesting a higher sensitivity to some drugs [144] and be useful as a guidance for treatment. Knowledge of DNA repair defects can be exploited in combination with additional drugs targeting other components of DNA repair pathways, such as olaparib, an inhibitor of PARP that participates in base-excision repair (BER), double-strand break repair by alt-EJ, repair of single-strand breaks (SSB) or chromatin epigenetic modifiers that will hypersensitize tumor cells to specific genotoxin treatment combinations [145]. Some of the nuclear kinases that are potential candidates for synthetic lethality are in Table 3.

### 6.1. Haspin and Aurora Kinases

Haspin and aurora B kinases phosphorylate histone H3 in mitotic progression [146]. Haspin inhibition with CHR-6494 impairs proliferation of breast cancer cell lines but have no effect on cell proliferation of breast cancer cell line xenographs [147]. Another haspin inhibitor, CX-6258 also impaired proliferation and generated the formation of micronuclei in melanoma cell lines [125]. In some cell lines, co-depletion of either p21 or p53 rescues the impairment in cell cycle progression caused haspin depletion [148]. Moreover, haspin elimination by CRISPR sensitizes tumor cells to VX680, an Aurora kinase B inhibitor in head and neck squamous cell carcinomas (HNSCC) and non-small cell lung cancer (NSCLC) cell lines [149]. 

However, the low specificity of haspin inhibitors [146,150], hinders studies based on synthetic lethality strategies. Within the Aurora serine/threonine kinase family, AURKA is involved in several processes, including the G2/M transition, mitotic spindle assembly and DNA replication [151]. The targeting of AURKA hypersensitize Myc overexpressing lymphoma cells to cyclophosphamide overcoming development of resistance to this drug [133]. Combined targeting of AURKA and WEE1 has a synergistic inhibition in head and neck squamous cell carcinoma xenographs by inhibiting tumor cell growth and extending animal survival [152]. 

AURKA inhibition is synthetic lethal in cells with *RB1* mutations [153]. AURKA inhibition is also synthetic lethal ARID1A-deficient colorectal cancer cells [154] and with CHEK1 kinase inhibitors in ovarian cancer [129]. AURKA inhibitor (MLN8237) is synthetic lethal with vincristine plus rituximab in aggressive non-Hodgkin lymphoma [132]. Alisertib, an AURKB inhibitor, is also a synthetic lethal inhibitor in combination with paclitaxel that interferes with microtubules in mitosis and detected in NSCLC [130], breast and ovarian cancer [131] in phase II studies.

### 6.2. VRK1

The VRK1 protein is a Ser-Thr chromatin kinase phosphorylates histone H3 in Thr3 [155,156] and H2A is Ser120 [157] and regulates transcription, proliferation and DNA damage responses [16,158]. High levels of VRK1 facilitate cell proliferation [159,160,161,162] and resistance to DNA damage treatments [122,155,158], which makes it a suitable potential target for cancer therapies. Knockdown screenings have identified VRK1 as a druggable kinase that will interfere with tumor cell viability and sensitizes cells to genotoxic drugs and radiation [52,123]. 

The structure of the VRK1 protein catalytic site predicts that this kinase not promiscuous and its inhibitors are likely to be highly specific [163,164]. Recently, some VRK1 specific inhibitors, based on an aminopyridine scaffold, are functional at pharmacological concentrations that inhibit tumor cell growth [165] and effect similar to VRK1 depletion [159], however, still need testing to determine their therapeutic potential.

An alternative approach is the targeting of two paralogs genes when their cooperation is necessary for cell viability. The initial evidence for this potential strategy resulted from the study of two members of the *VRK* gene family of Ser-Thr kinase in brain tumors [166]. These two genes can cooperate in loss of tumor cell viability when they are simultaneously impaired [166]. VRK2 is a paralog that shares its catalytic domain with VRK1 and which is anchored in the endoplasmic reticulum [167]. 

However, when VRK1 levels are low, there is an alternatively spliced isoform of VRK2, retaining its kinase domain, which is similar to VRK1 but lacks the endoplasmic reticulum anchor region and permits its translocation to nuclei where it can replace some of the VRK1 functions, such as phosphorylation of p53 [167]. In brain tumors, lacking VRK1, this compensatory mechanism is impaired by silencing the *VRK2* gene, and thus no VRK2 isoform can partially replace VRK1 [167], and cells become more sensitive to DNA damage [166].

### 6.3. PI3K (Phosphoinositide 3 Lipid Kinase (PI3K)-Related Protein Kinase) Family

The PI3K family includes three nuclear kinases associated with chromatin and DNA damage responses: ATM, ATR and DNA-PK [15]. These kinases are downstream of VRK1 in DDR [52,155,168,169].

ATM is a member of the PI3KK family implicated in the regulation of DNA damage responses [170]. ATM inhibitors, such as AZD0156 sensitize tumor cells to treatment with radiotherapy and is in phase I clinical trials (NCT02588105) with patients that have advanced stage solid tumors [170]. Two additional ATM inhibitors, AZD1390 in glioblastoma (NCT03423628) and M3541 in advanced and metastatic solid tumors (NCT03225105) are enrolling patients [170].

ATR has some new and selective inhibitors (M6620, M4344, AZD6738 and BAY1895344), that are in different stages of clinical development [171,172]. Another promising inhibitor, berzosertib (VX-970, M6620) has been tested in non-small cell lung cancer (NSCLC) patients in combination with gemcitabine showing good tolerance in phase 2 trial indicating that further studies are necessary [138]. VX-970 also enhances the sensitivity of NSCLC brain metastasis to radiation [173].

DNA-PK is a kinase implicated in DNA damage repair by the non-homologous end-joining pathway (NHEJ) of double-strand breaks. DNA-PK forms a complex and phosphorylates Ku70/80 at the DNA break-ends to be repaired [174,175,176]. DNA-PK belongs to the PI3K family. The inhibition of DNA-PK with AZD7648 sensitizes cells to doxorubicin and radiation induced DNA damage [141]. 

AZD7648 in ATM-deficient cells in combination with olaparib, a PARP inhibitor, increases genomic instability in PDX from breast cancer [141]. VX-984 inhibition of DNA-PK in patient-derived xenographs model sensitizes glioblastoma multiforme (GBM) to doxorubicin, and radiation treatments induced differentiation of GB stem cells by altering the stability of Sox2 leading to growth arrest [177,178]. Another DNA-PK inhibitor, M3814, has a similar sensitization effect [142].

CHEK1 and CHEK2 are direct downstream targets of ATR and ATM respectively, and consequently suitable targets for impairing the functions associated with them. CHEK1 inhibition with LY2603618 is synthetic lethal in combination with alisertib, an AURKA inhibitor, in ovarian cancer cells by sensitizing them to platinum and inducing apoptosis [129].

## 7. Conclusions

Epigenetic manipulation through the combined targeting of histone modification enzymes and nuclear kinases, which is implicated in DNA damage responses in cancer treatment, should aim to sensitize tumor cells to known effective drugs, such as those based on induction of DNA damage treatment and/or facilitate the host response to treatments based on immunotherapy. Considering the potential effects of different epigenetic inhibitors that target chromatin, it is likely that they can play different roles depending on the specific type of cancer and its stage.

Combinations of new drugs targeting alternative pathways implicated in chromatin remodeling with current genotoxic treatments at lower doses can improve cancer treatment leading to a reduced toxicity and increased immunogenicity. This, combined with an improved diagnosis, can result in longer disease-free periods with a better quality of life.

## Figures and Tables

**Table 2:** Candidate combinations for synthetic lethality strategies targeting chromatin deacetylases (HDAC) and acetylases (KAT).

Targets (HDAC or KAT)		Inhibitor Combinations	Tumor	Ref.
1	**HDAC**	panobinostat	Ovarian cancer cell line	[78]
	PARP	olaparib		
2	**HDAC**	panobinostat	Multiple myeloma	[79]
	Proteasome	Bortezomid		
3	**HDAC**	panobinostat	glioblastoma	[80]
	BRD	OTX015		
4	**HDAC**	Panobinostat, Pracinostat, Entinostat, Vorinostat, Belinostat	MDS, AML, CML, lymphomas, NSCLC, breast cancer, multiple cancer types	[81]
	DNMT	Azacitidine/Decitabine		
5	**Tip60 (KAT5)**	MG149	Lung cane cell lines	[52]
	DNA damage	doxorubicin		
6	**P300 (KAT3B)**	C646	Melanoma with BRAF(V600E)	[82]
	BRAF	Vemurafenib/ AZ628		

**Table 3:** Nuclear kinases that are druggable for synthetic lethality strategies.

Kinase Targeted	Kinase Inhibition	Drug Combination	Tumor Type	Ref
**VRK1**	depletion	Doxorubicin, radiation	Lung, sarcoma, glioma and breast cancer cell lines	[122,123,124]
	depletion	temozolomide	Glioblastoma cell lines	[123]
	depletion	Radiation, Olaparib	Glioblastoma cell lines	[123,124]
**Haspin**	CX-6258 CHR-6494		Melanoma	[125,126]
**Aurora B**	VX-680 GSK1070916	Imatinib resistance	Lung and breast cancer cell lines.	[127,128]
**Aurora A**	alisertib	LY2603618 (CHEK1 inhi.), Paclitaxel	Ovary, breast, SCLC	[129,130,131]
**Aurora A**	MLN8237	Vincristine + rituximab	Non-Hodgkin lymphoma	[132]
	MLN8237	cyclophosphamide	Myc-overeexpressing lymphomas	[133]
**PLK1**	volasertib		Breast cancer palciclobib resistance	[134,135]
**ATM**	KU55933 KU60019 AZ 20 CGK733	Radiation	Bladder Neuroblastoma	[136]
**NBS1-ATM**	Mirin	Cis-platin	ovarian	[137]
**ATR**	AZ20 ETP-46464 Berzosertib Ceralasertib CGK733	radiation Olaparib	- Lung cancer Metastatic melanoma, Ovarian resistant to PARP inhibitors	[138,139,140]
**DNA-PK**	Peposertib (M3814) AZD7648	- Olaparib	Rectal cancer	[141,142]
**CDK4/6**	trilaciclib	Platinum/etoposide topotecan	ES-SCLC	[143]
